# Meta-evaluation of meta-analysis: ten appraisal questions for biologists

**DOI:** 10.1186/s12915-017-0357-7

**Published:** 2017-03-03

**Authors:** Shinichi Nakagawa, Daniel W. A. Noble, Alistair M. Senior, Malgorzata Lagisz

**Affiliations:** 1 Evolution & Ecology Research Centre and School of Biological, Earth and Environmental Sciences, University of New South Wales, Sydney, NSW 2052 Australia; 2 Diabetes and Metabolism Division, Garvan Institute of Medical Research, 384 Victoria Street, Darlinghurst, Sydney, NSW 2010 Australia; 3 Charles Perkins Centre, University of Sydney, Sydney, NSW 2006 Australia; 4 School of Mathematics and Statistics, University of Sydney, Sydney, NSW 2006 Australia

**Keywords:** Effect size, Biological importance, Non-independence, Meta-regression, Meta-research, Publication bias, Quantitative synthesis, Reporting bias, Statistical significance, Systematic review

## Abstract

Meta-analysis is a statistical procedure for analyzing the combined data from different studies, and can be a major source of concise up-to-date information. The overall conclusions of a meta-analysis, however, depend heavily on the quality of the meta-analytic process, and an appropriate evaluation of the quality of meta-analysis (meta-evaluation) can be challenging. We outline ten questions biologists can ask to critically appraise a meta-analysis. These questions could also act as simple and accessible guidelines for the authors of meta-analyses. We focus on meta-analyses using non-human species, which we term ‘biological’ meta-analysis. Our ten questions are aimed at enabling a biologist to evaluate whether a biological meta-analysis embodies ‘mega-enlightenment’, a ‘mega-mistake’, or something in between.

## Meta-analyses can be important and informative, but are they all?

Last year saw 40 years since the coining of the term ‘meta-analysis’ by Gene Glass in 1976 [1, 2]. Meta-analyses, in which data from multiple studies are combined to evaluate an overall effect, or effect size, were first introduced to the medical and social sciences, where humans are the main species of interest [3–5]. Decades later, meta-analysis has infiltrated different areas of biological sciences [6], including ecology, evolutionary biology, conservation biology, and physiology. Here non-human species, or even ecosystems, are the main focus [7–12]. Despite this somewhat later arrival, interest in meta-analysis has been rapidly increasing in biological sciences. We have argued that the remarkable surge in interest over the last several years may indicate that meta-analysis is superseding traditional (narrative) reviews as a more objective and informative way of summarizing biological topics [8].

It is likely that the majority of us (biologists) have never conducted a meta-analysis. Chances are, however, that almost all of us have read at least one. Meta-analysis can not only provide quantitative information (such as overall effects and consistency among studies), but also qualitative information (such as dominant research trends and current knowledge gaps). In contrast to that of many medical and social scientists [3, 5], the training of a biologist does not typically include meta-analysis [13] and, consequently, it may be difficult for a biologist to evaluate and interpret a meta-analysis. As with original research studies, the quality of meta-analyses vary immensely. For example, recent reviews have revealed that many meta-analyses in ecology and evolution miss, or perform poorly, several critical steps that are routinely implemented in the medical and social sciences [14, 15] (but also see [16, 17]).

**Fig. 1.**
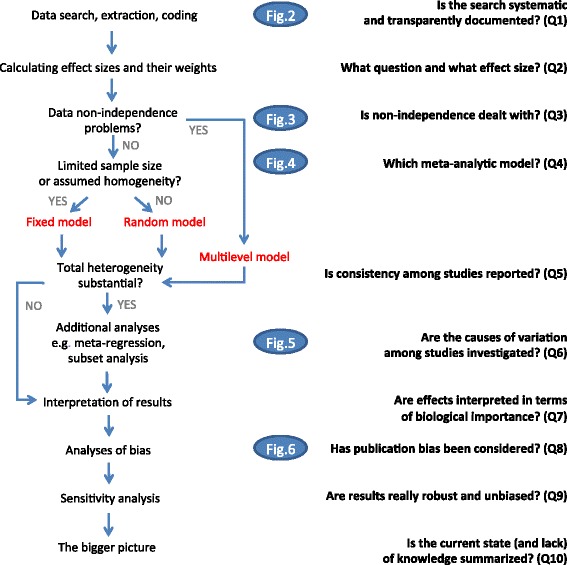
Mapping the process (on the *left*) and main evaluation questions (on the *right*) for meta-analysis. References to the relevant figures (Figs. 2, 3, 4, 5 and 6) are included in the *blue ovals*

The aim of this review is to provide ten appraisal questions that one should ask when reading a meta-analysis (cf., [18, 19]), although these questions could also be used as simple and accessible guidelines for researchers conducting meta-analyses. In this review, we only deal with ‘narrow sense’ or ‘formal’ meta-analyses, where a statistical model is used to combine common effect sizes across studies, and the model takes into account sampling error, which is a function of sample size upon which each effect size is based (more details below; for discussions on the definitions of meta-analysis, see [15, 20, 21]). Further, our emphasis is on ‘biological’ meta-analyses, which deal with non-human species, including model organisms (nematodes, fruit flies, mice, and rats [22]) and non-model organisms, multiple species, or even entire ecosystems. For medical and social science meta-analyses concerning human subjects, large bodies of literature and excellent guidelines already exist, especially from overseeing organizations such as the Cochrane (Collaboration) and the Campbell Collaboration. We refer to the literature and the practices from these ‘experienced’ disciplines where appropriate. An overview and roadmap of this review is presented in Fig. 1. Clearly, we cannot cover all details, but we cite key references in each section so that interested readers can follow up.

## Q1: Is the search systematic and transparently documented?

When we read a biological meta-analysis, it used to be (and probably still is) common to see a statement like “a comprehensive search of the literature was conducted” without mention of the date and type of databases the authors searched. Documentation on keyword strings and inclusion criteria is often also very poor, making replication of search outcomes difficult or impossible. Superficial documentation also makes it hard to tell whether the search really was comprehensive, and, more importantly, systematic.

A comprehensive search attempts to identify (almost) all relevant studies/data for a given meta-analysis, and would thus not only include multiple major databases for finding published studies, but also make use of various lesser-known databases to locate reports and unpublished studies. Despite the common belief that search results should be similar among major databases, overlaps can sometimes be only moderate. For example, overlap in search results between *Web of Science* and *Scopus* (two of the most popular academic databases) is only 40–50% in many major fields [23]. As well as reading that a search is comprehensive, it is not uncommon to read that a search was systematic. A systematic search needs to follow a set of pre-determined protocols aimed at minimizing bias in the resulting data set. For example, a search of a single database, with pre-defined focal questions, search strings, and inclusion/exclusion criteria, can be considered systematic, negating some bias, though not necessarily being comprehensive. It is notable that a comprehensive search is preferable but not necessary (and often very difficult to do) whereas a systematic search is a must [24].

**Fig. 2.**
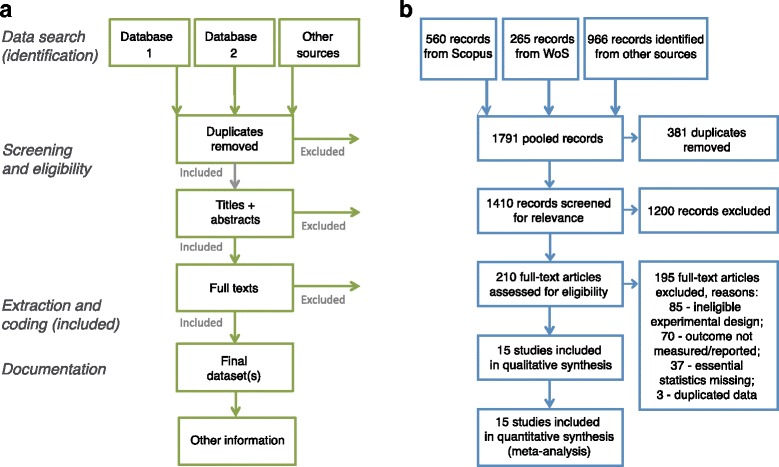
Preferred Reporting Items for Systematic Reviews and Meta-Analyses. (PRISMA). **a** The main components of a systematic review or meta-analysis. The data search (identification) stage should, ideally, be preceded by the development of a detailed study protocol and its preregistration. Searching at least two literature databases, along with other sources of published and unpublished studies (using backward and forward citations, reviews, field experts, own data, grey and non-English literature) is recommended. It is also necessary to report search dates and exact keyword strings. The screening and eligibility stage should be based on a set of predefined study inclusion and exclusion criteria. Criteria might differ for the initial screening (title, abstract) compared with the full-text screening, but both need to be reported in detail. It is good practice to have at least two people involved in screening, with a plan in place for disagreement resolution and calculating disagreement rates. It is recommended that the list of studies excluded at the full-text screening stage, with reasons for their exclusion, is reported. It is also necessary to include a full list of studies included in the final dataset, with their basic characteristics. The extraction and coding (included) stage may also be performed by at least two people (as is recommended in medical meta-analysis). The authors should record the figures, tables, or text fragments within each paper from which the data were extracted, as well as report intermediate calculations, transformations, simplifications, and assumptions made during data extraction. These details make tracing mistakes easier and improve reproducibility. Documentation should include: a summary of the dataset, information on data and study details requested from authors, details of software used, and code for analyses (if applicable). **b** It is now becoming compulsory to present a PRISMA diagram, which records the flow of information starting from the data search and leading to the final data set. *WoS Web of Science*

For most meta-analyses in medicine and social sciences, the search steps are systematic and well documented for reproducibility. This is because these studies follow a protocol named the PRISMA (Preferred Reporting Items for Systematic Reviews and Meta-Analyses) statement [25, 26]; note that a meta-analysis should usually be a part of a systematic review, although a systematic review may or may not include meta-analysis. The PRISMA statement facilitates transparency in reporting meta-analytic studies. Although it was developed for health sciences, we believe that the details of the four key elements of the PRISMA flow diagram (‘identification’, ‘screening’, ‘eligibility’, and ‘included’) should also be reported in a biological meta-analysis [8]. Figure 2 shows: A) the key ideas of the PRISMA statement, which the reader should compare with the content of a biological meta-analysis; and B) an example of a PRISMA diagram, which should be included as part of meta-analysis documentation. The bottom line is that one should assess whether search and screening procedures are reproducible and systematic (if not comprehensive; to minimize potential bias), given what is described in the meta-analytic paper [27, 28].

## Q2: What question and what effect size?

A meta-analysis should not just be descriptive. The best meta-analyses ask questions or test hypotheses, as is the case with original research. The meta-analytic questions and hypotheses addressed will generally determine the types of effect size statistics the authors use [29–32], as we explain below. Three broad groups of effect size statistics are based on are: 1) the difference between the means of two groups (for example, control versus treatment); 2) the relationship, or correlation, between two variables; and 3) the incidence of two outcomes (for example, dead or alive) in two groups (often represented in a 2 by 2 contingency table); see [3, 7] for comprehensive lists of effect size statistics. Corresponding common effect size statistics are: 1) standardized mean difference (SMD; often referred to as *d*, Cohen’s *d*, Hedges’ *d* or Hedges’ *g*) and the natural logarithm (log) of the response ratio (denoted as either ln*R* or ln*RR* [33]); 2) Fisher’s *z*-transformed correlation coefficient (often denoted as *Zr*); and 3) the natural logarithm of the odds ratio (ln*OR*) and relative risk (ln*RR*; not to be confused with the response ratio).

We have also used and developed methods associated with less common effect size statistics such as log hazard ratio (ln*HR*) for comparing survival curves [34–37], and also the log coefficient of variation ratio (ln*CVR*) for comparing differences between the variances, rather than means, of two groups [38–40]. It is important to assess whether a study used an appropriate effect size statistic for the focal question. For example, when the authors are interested in the effect of a certain treatment, they should typically use SMD or response ratio, rather than *Zr*. Most biological meta-analyses will use one of the standardized effect sizes mentioned above. These effect sizes are referred to as standardized because they are unit-less (dimension-less), and thus are comparable across studies, even if those studies use different units for reporting (for example, size can be measured by weight [g] or length [cm]). However, unstandardized effect sizes (raw mean difference or regression coefficients) can be used, as happens in medical and social sciences, when all studies use common and directly comparable units (for example, blood pressure [mmHg]).

That being said, a biological meta-analysis will often bring together original studies of different types (such as combinations of experimental and observational studies). As a general rule, SMD is considered a better fit for experimental studies, whereas *Zr* is better for observational (correlational) studies. In some cases different effect sizes might be calculated for different studies in a meta-analysis and then be converted to a common type prior to analysis: for example, *Zr* and SMD (and also ln*OR*) are inter-convertible. Thus, if we were, for example, interested in the effect of temperature on growth, we could combine results from experimental studies that compare mean growth at two temperatures (SMD) with results from observational studies that compare growth across a temperature gradient (*Zr*) in a single meta-analysis by transforming SMD from experimental studies to *Zr* [29–32].

## Q3: Is non-independence taken into account?

Statistical non-independence occurs when data points (in this case, effect sizes) are somewhat related to each other. For example, multiple effect sizes may be taken from a single study, making such effect sizes correlated. Failing to account for non-independence among effect sizes (or data points) can lead to erroneous conclusions [14, 41–44]—typically, an invalid conclusion of statistical significance (type I error; also see Q7). Many authors do not correct for non-independence (see [15]). There are two main reasons for this: the authors may be unaware of non-independence among effect sizes or they may have difficulty in appropriately accounting for the correlated structure despite being aware of the problem.

**Fig. 3.**
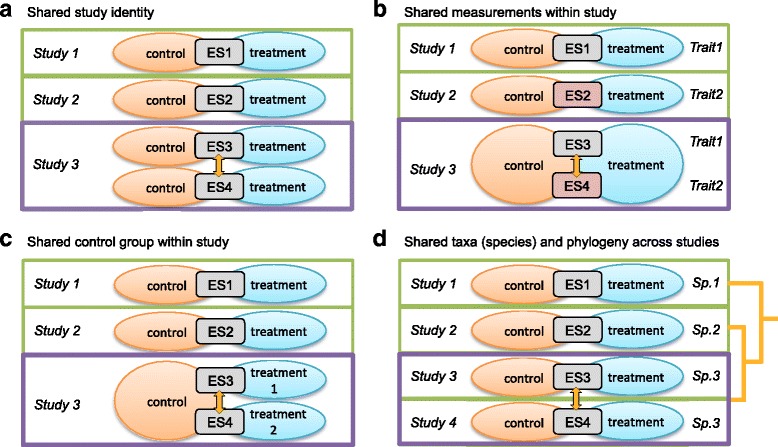
Common sources of non-independence in biological meta-analyses. **a**–**d** Hypothetical examples of the four most common scenarios of non-independence (**a**-**d**). Orange lines and *arrows* indicate correlations between effect sizes. Effect size estimate (*gray boxes*, ‘*ES*’) is the ratio of (or difference between) the means of two groups (control versus treatment). Scenarios **a**, **b**, and **d** may apply to other types of effect sizes (e.g., correlation), while scenario **c** is unique to situations where two or more groups are compared to one control group. **a** Multiple effect sizes can be calculated from a single study. Effect sizes in study 3 are not independent of each other because effects (ES3 and ES4) are derived from two experiments using samples from the same population. For example, a study exposed females and males to increased temperatures, and the results are reported separately for the two sexes. **b** Effect sizes taken from the same study (study 3) are derived from different traits measured from the same subjects, resulting in correlations among these effect sizes. For example, body mass and body length are both indicators of body size, with studies 1 and 2 reporting just one of these measurements and study 3 reporting both for the same group of individuals. **c** Effect sizes can be correlated via contrast with a common ‘control’ group of individuals; for example, both effect sizes from study 3 share a common control treatment. A study may, for example, compare a balanced diet (control) with two levels of a protein-enriched diet. **d** In a multi-species study effect sizes can be correlated when they are based on data from organisms from the same taxonomic unit, due to evolutionary history. Effect sizes taken from studies 3 and 4 are not independent, because these studies were performed on the same species (*Sp.3*). Additionally, all species share a phylogenetic history, and thus all effect sizes can be correlated with one another in accordance with time since evolutionary divergence between species

To help the reader to detect non-independence where the authors have failed to take it into account, we have illustrated four common types of dependent effect sizes in Fig. 3, with the legend including a biological example for each type. Phylogenetic relatedness (Fig. 3d) is unique to biological meta-analyses that include multiple species [14, 42, 45]. Correction for phylogenetic non-independence can now be implemented in several mainstream software packages, including *metafor* [46].

Where non-independence goes uncorrected because of the difficulty of appropriately accounting for the correlated structure, it is usually because the non-independence is incompatible with the two traditional meta-analytic models (the fixed-effect and the random-effects models—see Q4) that are implemented in widely used software (for example, *Metawin* [47]). Therefore, it was (and still is) common to see averaging of non-independent effect sizes or the selection of one among several related effect sizes. These solutions are not necessarily incorrect (see [48]), but may be limiting, and clearly lead to a loss of information [14, 49]. The reader should be aware that it is preferable to model non-independence directly by using multilevel meta-analytic models (see Q4) if the dataset contains a sufficient number of studies (complex models usually require a large sample size) [14].

## Q4: Which meta-analytic model?

**Fig. 4.**
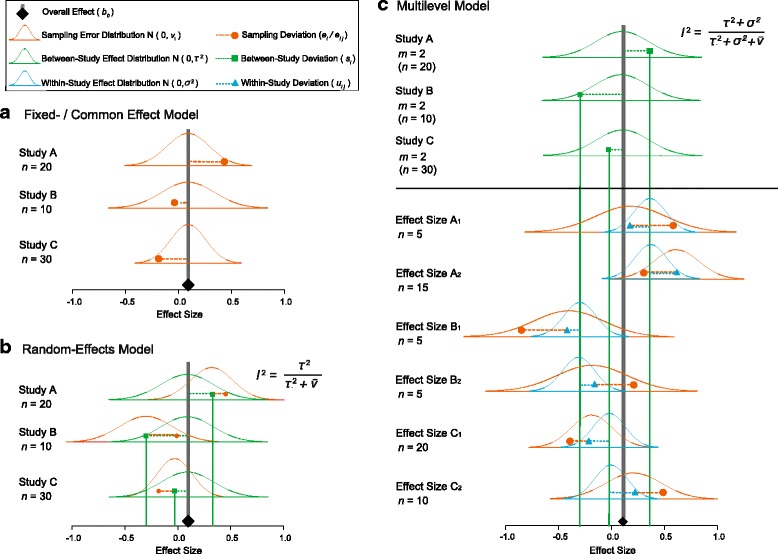
Visualizations of the three main types of meta-analytic models and their assumptions. **a** The fixed-effect model can be written as *y*_*i*_ = *b*_0_ + *e*_*i*_, where *y*_*i*_ is the observed effect for the *i*th study (*i* = 1…*k*; *orange circles*), *b*_0_ is the overall effect (overall mean; *thick grey line* and *black diamond*) for all *k* studies and *e*_*i*_ is the deviation from *b*_0_ for the *i*th study (*dashed orange lines*), and *e*_*i*_ is distributed with the sampling variance *ν*_*i*_ (*orange curves*); note that this variance is sometimes called within-study variance in the literature, but we reserve this term for the multilevel model below. **b** The random-effects model can be written as *y*_*i*_ = *b*_0_ + *s*_*i*_ + *e*_*i*_, where *b*_0_ is the overall mean for different studies, each of which has a different study-specific mean (*green squares* and *green solid lines*), deviating by *s*_*i*_ (*green dashed lines*) from *b*_0_, *s*_*i*_ is distributed with a variance of *τ* ^2^ (the between-study variance; *green curves*); note that this is the conventional notation for the between-study variance, but in a biological meta-analysis, it can be referred to as, say, *σ* ^2^_[study]_. The other notation is as above. Displayed on the *top-right* is the formula for the heterogeneity statistic, *I* ^2^ for the random-effects model, where $\overline{v}$ is a typical sampling variance (perhaps, most easily conceptualized as the average value of sampling variances, *ν*_*i*_). **c** The simplest multilevel model can be written as *y*_*ij*_ = *b*_0_ + *s*_*i*_ + *u*_*ij*_ + *e*_*ij*_, where *u*_*ij*_ is the deviation from *s*_*i*_ for *j*th effect size for the *i*th study (*blue triangles* and *dashed blue lines*) and is distributed with the variance of *σ* ^2^ (the within-study variance or it may be denoted as *σ* ^2^_[effect size]_; *blue curves*), *e*_*ij*_ is the deviation from *u*_*ij*_, and the other notations are the same as above. Each of *k* studies has *m* effect sizes (*j* = 1…*m*). Displayed on the *top-right* is the multilevel meta-analysis formula for the heterogeneity statistic, *I* ^2^, where both the numerator and denominator include the within-study variance, *σ* ^2^, in addition to what appears in the formula for the random-effects model

There are three main kinds of meta-analytic models, which differ in their assumptions about the data being analyzed, but for all three the common and primary goal is to estimate an overall effect (but see Q5). These models are: i) fixed-effect models (also referred to as common-effect models [31]); ii) random-effects models [50]; and iii) multilevel (hierarchical) models [14, 49]. We have depicted these three kinds of models in Fig. 4. When assessing a meta-analysis, the reader should be aware of the different assumptions each model makes. For the fixed-effect (Fig. 4a) and random-effects (Fig. 4b) models, all effect sizes are assumed to be independent (that is, one effect per study, with no other sources of non-independence; see Q3). The other major assumption of a fixed-effect model is that all effect sizes share a common mean, and thus that variation among data is solely attributable to sampling error (that is, the sampling variance, *v*_*i*_, which is related to the sample size for each effect size; Fig. 4a). This assumption, however, is unrealistic for most biological meta-analyses (see [22]), especially those involving multiple populations, species, and/or ecosystems [14, 51]. The use of a fixed-effect model could be justified where the effect sizes are obtained from the same species or population (assuming one effect per study and that the effect sizes are independent of each other). Random-effects models relax the assumption that all studies are based on samples from the same underlying population, meaning that these models can be used when different studies are likely to quantify different underlying mean effects (for example, one study design yields a different effect than another), as is likely to be the case for a biological meta-analysis (Fig. 4b). A random-effects model needs to quantify the between-study variance, *τ* ^2^, and to estimate this variance correctly requires a sample size of perhaps over ten effect sizes. Thus, random-effects models may not be appropriate for a meta-analysis with very few effect sizes, and fixed-effect models may be appropriate in such situations (bearing in mind the aforementioned assumptions). Multilevel models relax the assumptions of independence made by fixed-effect and random-effects models; that is, for example, these models allow for multiple effect sizes to come from the same study, which may be the case if one study contains several different experimental treatments, or the same experimental treatment is applied across species within one study. The simplest multilevel model depicted in Fig. 4c includes study effects, but it is probably not difficult to imagine this multilevel approach being extended to incorporate more ‘levels’, such as species effects, as well (for more details see [13, 14, 41, 45, 49, 51–54]; incorporating the types of non-independence described in Fig. 3b–d requires modeling of correlation and covariance matrices).

**Fig. 5.**
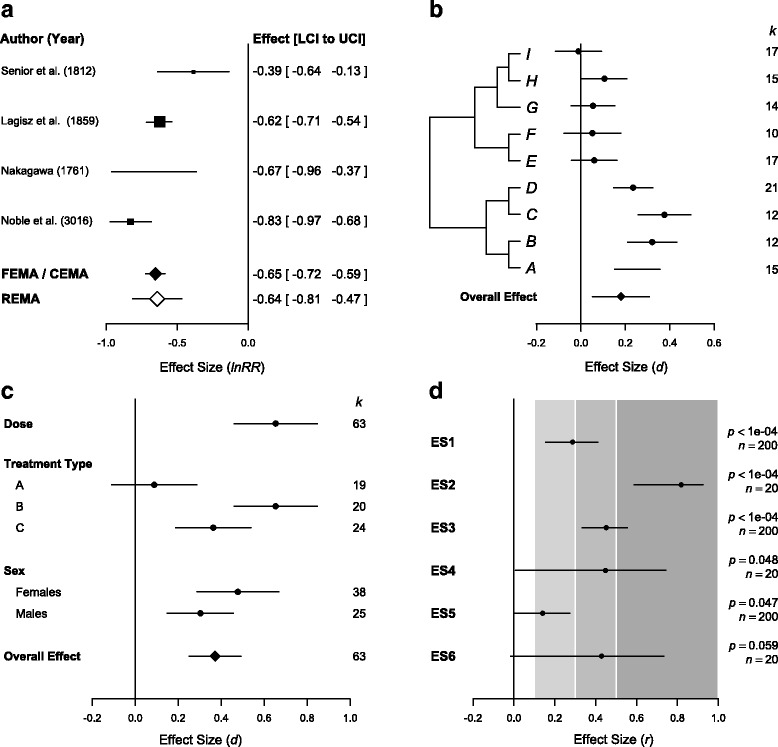
Examples of forest plots used in a biological meta-analysis to represent effect sizes and their associated precisions. **a** A conventional forest plot displaying the magnitude and uncertainty (95% confidence interval, CI) of each effect size in the dataset, as well as reporting the associated numerical values and a reference to the original paper. The sizes of the shapes representing point estimates are usually scaled based on their precision (1/Standard error). *Diamonds* at the bottom of the plot display the estimated overall mean based on both fixed-effect meta-analysis/‘common-effect’ meta-analysis (*FEMA/CEMA*) and random-effects meta-analysis (*REMA*) models. **b** A forest plot that has been augmented to display a phylogenetic relationship between different taxa in the analysis; the estimated *d* seems on average to be higher in some clades than in the others. A *diamond* at the bottom summarizes the aggregate mean as estimated by a multi-level meta-analysis accounting for the given phylogenetic structure. On the right is the number of effect sizes for each species (*k*), although similarly one could also display the number of individuals/sample-size (*n*), where only one effect size per species is included. **c** As well as displaying overall effect (*diamond*), forest plots are sometimes used to display the mean effects from different sub-groups of the data (e.g., effects separated by sex or treatment type), as estimated with data sub-setting or meta-regression, or even a slope from meta-regression (indicating how an effect changes with increasing continuous variable, e.g., dosage). **d** Different magnitudes of correlation coefficient (*r*), and associated 95% CIs, *p* values, and the sample size on which each estimate is based. The space is shaded according to effect magnitude based on established guidelines; *light grey*, *medium grey*, and *dark grey* correspond to small, medium, and large effects, respectively

It is important for you, as the reader, to check whether the authors, given their data, employed an appropriate model or set of models (see Q3), because results from inappropriate models could lead to erroneous conclusions. For example, applying a fixed effect model, when a random effects model is more appropriate, may lead to errors in both the estimated magnitude of the overall effect and its uncertainty [55]. As can be seen from Fig. 4, each of the three main meta-analytical models assume that effect sizes are distributed around an overall effect (*b*_*0*_). The reader should also be aware that this estimated overall effect (meta-analytic mean) is most commonly presented in an accompanying forest plot(s) [22, 56, 57]. Figure 5a is a forest plot of the kind that is typically seen in medical and social sciences, with both overall means from the fixed-effect or the common effect meta-analysis (FEMA/CEMA) model, and the random-effects meta-analysis (REMA) model. In a multiple-species meta-analysis, you may see an elaborate forest plot such as that in Fig. 5b.

## Q5: Is the level of consistency among studies reported?

The overall effect reported by a meta-analysis cannot be properly interpreted without an analysis of the heterogeneity, or inconsistency, among effect sizes. For example, an overall mean of zero can be achieved when effect sizes are all zero (homogenous; that is, the between-study variance is 0) or when all effect sizes are very different (heterogeneous; the between study variance is >0) but centered on zero, and clearly one should draw different conclusions in each case. Rather disturbingly, we have recently found that in ecology and evolutionary biology, tests of heterogeneity and their corresponding statistics (*τ* ^2^, *Q*, and *I* ^2^) are only reported in about 40% of meta-analyses [58]. Cochran’s *Q* (often referred to as *Q*_*total*_ or *Q*_*T*_) is a test statistic for the between-study variance (*τ* ^2^), which allows one to assess whether the estimated between-study variance is non-zero (in other words, whether a fixed-effect model is appropriate as this model assumes *τ* ^2^ = 0) [59]. As a test statistic, *Q* is often presented with a corresponding *p* value, which is interpreted in the conventional manner. However, if presented without the associated *τ* ^2^, *Q* can be misleading because, as is the case with most statistical tests, *Q* is more likely to be significant when more studies are included even if *τ* ^2^ is relatively small (see also Q7); the reader should therefore check whether both statistics are presented. Having said that, the magnitude of the between-study variance (*τ* ^2^) can be hard to interpret because it is dependent on the scale of the effect size. The heterogeneity statistic, *I* ^2^, which is a type of intra-class correlation, has also been recommended as it addresses some of the issues associated with *Q* and *τ* ^2^ [60, 61]. *I* ^2^ ranges from 0 to 1 (or 0 to 100%) and indicates how much of the variation in effect sizes is due to the between-study variance (*τ* ^2^; Fig. 4b) or, more generally, the proportion of variance not attributable to sampling (error) variance ($\overline{v}$; see Fig. 4b, c; for more details and extensions, see [13, 14, 49, 58]). Tentatively suggested benchmarks for *I* ^2^ are low, medium, and high heterogeneity of 25, 50, and 75% [61]. These values are often used in meta-analyses in medical and social sciences for interpreting the degree of heterogeneity [62, 63]. However, we have shown that the average *I* ^2^ in meta-analyses in ecology and evolution may be as high as 92%, which may not be surprising as these meta-analyses are not confined to a single species (or human subjects) [58]. Accordingly, the reader should consider whether these conventional benchmarks are applicable to the biological meta-analysis under consideration. The quantification and reporting of heterogeneity statistics is essential for any meta-analysis, and you need to make sure some or combinations of these three statistics are reported in a meta-analysis before making generalisations based on the overall mean effect (except when using fixed-effect models).

## Q6: Are the causes of variation among studies investigated?

After quantifying variation among effect sizes beyond sampling variation (*I* ^2^ ), it is important to understand the factors, or moderators, that might explain this additional variation, because it can elucidate important processes mediating variation in the strength of effect. Moderators are equivalent to explanatory (independent) variables or predictors in a normal linear model [8, 49, 62]. For example, in a meta-analysis examining the effect of experimentally increased temperature on growth using SMD (control versus treatment comparison) studies might vary in the magnitude of temperature increase: say 10 versus 20 °C in the first study, but 12 versus 16 °C in the second. In this case, the moderator of interest is the temperature difference between control and treatment groups (10 °C for the first study and 4 °C for the second). This difference in study design may explain variation in the magnitude of the observed effect sizes (that is, the SMD of growth at the two temperatures). Models that examine the effects of moderators are referred to as meta-regressions. One important thing to note is that meta-regression is just a special type of weighted regression. Therefore, the usual standard practices for regression analysis also apply to meta-regression. This means that, as a reader, you may want to check for the inclusion of too many predictors/moderators in a single model, or ‘over-fitting’ (the rule of thumb is that the authors may need at least ten effect sizes per estimated moderator) [64], and for ‘fishing expeditions’ (also known as ‘data dredging’ or ‘*p* hacking’; that is, non-hypothesis-based exploration for statistical significance [28, 65, 66]).

**Fig. 6.**
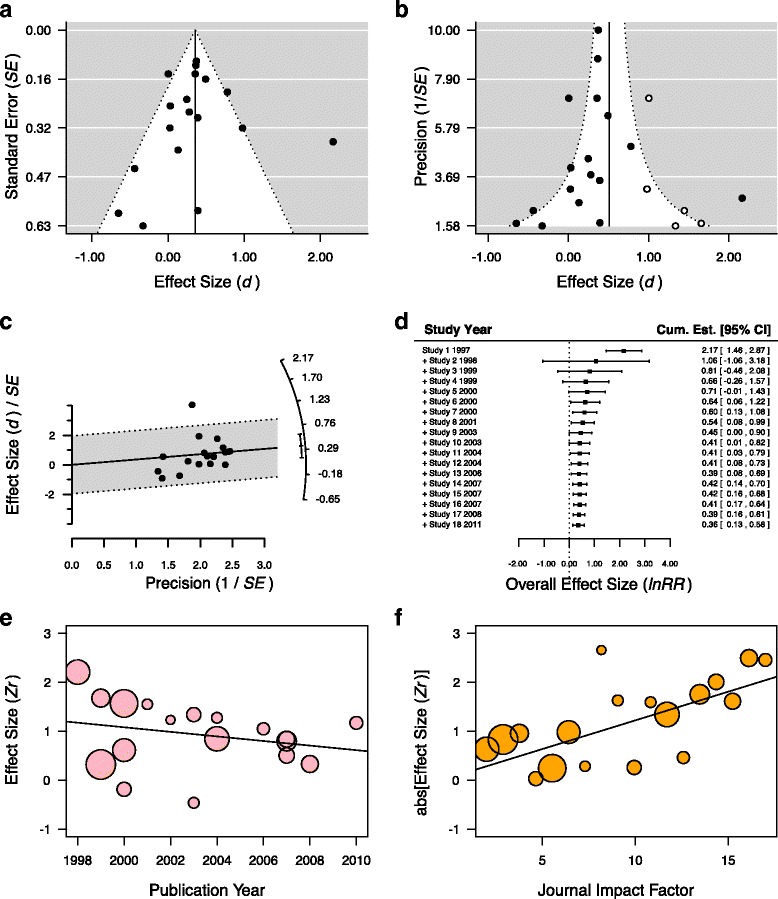
Graphical assessment tools for testing for publication bias. **a** A funnel plot showing greater variance among effects that have larger standard errors (*SE*) and that are thus more susceptible to sampling variability. Some studies in the lower right corner of the plot, opposite to most major findings, with large SE (less likely to detect significant results) are potentially missing (not shown), suggesting publication bias. **b** Often funnel plots are depicted using precision (1/SE), giving a different perspective of publication bias, where studies with low precision (or large SE) are expected to show greater sampling variability compared to studies with high precision (or low SE). Note that the data in panel **b** are the same as in panel **a**, except that a trim-and-fill analysis has been performed in **b**. A trim-and-fill analysis estimates the number of studies missing from the meta-analysis and creates ‘mirrored’ studies on the opposite side of the funnel (*unfilled dots*) to estimate how the overall effect size estimate is impacted by these missing studies. **c** Radial (Galbraith) plot in which the slope should be close to zero, if little publication bias exists, indicating little asymmetry in a corresponding funnel plot (compare it with **b**); radial plots are closely associated with Egger’s tests. **d** Cumulative meta-analysis showing how the effect size changes as the number of studies on a particular topic increases. In this situation, the addition of effect size estimates led to convergence on an overall estimate of 0.36, and the confidence intervals decrease as the precision of the estimate increases. **e** Bubble plot showing a temporal trend in effect size (*Zr*) across years. Here effect sizes are weighted by their precision; larger bubbles indicate more precise estimates and smaller bubbles less precise. **f** Bubble plot of the relationship between effect size and impact factors of journals, indicating that larger magnitudes of effect sizes (the absolute values of *Zr*) tend to be published in higher impact journals

Moderators can be correlated with each other (that is, be subject to the multicollinearity problem) and this dependence, in turn, could lead authors to attribute an effect to the wrong moderator [67]. For example, in the aforementioned meta-analysis of temperature on growth, the study may claim that females grew faster than males when exposed to increased temperatures. However, if most females came from studies where higher temperature increases were used but males were usually exposed to small increases, the moderators for sex and temperature would be confounded. Accordingly, the effect may be due to the severity of the temperature change rather than a sex effect. Readers should check whether the authors have examined potential confounding effects of moderators and reported how different potential moderators are related to one another. It is also important to know the sources of the moderator data; for example, species-specific data can be obtained from sources (papers, books, databases) other than the primary studies from which effect sizes were taken (Q1). Meta-regression results can be presented in a forest plot, as in Fig. 5c (see also Q6 and Fig. 6e, f; the standardization of moderators may often be required for analyzing moderators [68]).

Another way of exploring heterogeneity is to run separate meta-analysis on data subsets (for example, separating effect sizes by the sex of exposed animals). This is similar to running a meta-regression with categorical moderators (often referred to as subgroup analysis), with the key difference being that the authors can obtain heterogeneity statistics (such as *I* ^2^) for each subset in a subset analysis [69]. It is important to note that many meta-analytic studies include more than one meta-analysis, because several different types of data are included, even though these data pertain to one topic (for example, the effect of increased temperature not only on body growth, but also on parasite load). You, as a reader, will need to evaluate whether the authors’ sub-grouping or sub-setting of their data makes sense biologically; hopefully the authors will have provided clear justification (Q1).

## Q7: Are effects interpreted in terms of biological importance?

Meta-analyses should focus on biological importance (which is reflected in estimated effects and their uncertainties) rather than on *p* values and statistical significance, as is outlined in Fig. 5d [29, 70–72]. It should be clear to most readers that interpreting results only in terms of statistical significance (*p* values) can be misleading. For example, in terms of effects’ magnitudes and uncertainties, ES4 and ES6 in Fig. 5d are nearly identical, yet ES4 is statistically significant, while ES6 is not. Also, ES1–3 are all what people describe as ‘highly significant’, but their magnitudes of effect, and thus biological relevance, are very different. The term ‘effective thinking’ is used to refer to the philosophy of placing emphasis on the interpretation of overall effect size in terms of biological importance rather than statistical significance [29]. It is useful for the reader to know that each of ES1–3 in Fig. 5d can be classified as what Jacob Cohen proposed as small, medium, and large effects, which are *r* = 0.1, 0.3, and 0.5, respectively [73]; for SMD, corresponding benchmarks are *d* (SMD) = 0.2, 0.5, and 0.8 [29, 61]. Researchers may have good intuition for the biological relevance of a particular *r* value, but this may not be the case for SMD. Thus, it may be helpful to know that Cohen’s benchmarks for *r* and *d* are comparable. Having said that, these benchmarks, along with those for *I* ^2^, have to be used carefully, because what constitute biologically important effect magnitudes can vary according to the biological questions and systems (for example, 1% difference in fitness would not matter in ecological time but it certainly does over evolutionary time). We stress that authors should primarily be discussing their effect sizes (point estimates) and uncertainties in terms of point estimates (confidence intervals, or credible intervals, CIs) [29, 70, 72]. Meta-analysts can certainly note statistical significance, which is related to CI width, but direct description of precision may be more useful. Note that effect magnitude and precision are exactly what are displayed in forest plots (Fig. 5).

## Q8: Has publication bias been considered?

Meta-analysts have to assume that research is published regardless of statistical significance, and that authors have not selectively reported results (that is, that there is no publication bias and no reporting bias) [74–76]. This is unlikely. Therefore, meta-analysts should check for publication bias using statistical and graphical tools. The reader should know that the commonly used methods for assessing publication bias are funnel plots (Fig. 6a, b), radial (Galbraith) plots (Fig. 6c), and Egger’s (regression) tests [57, 77, 78]; these methods visually or statistically (Egger’s test) help to detect funnel asymmetry, which can be caused by publication bias [79]. However, you should also know that funnel asymmetry may be an artifact of too few a number of effect sizes. Further, funnel asymmetry can result from heterogeneity (non-zero between-study variance, *τ* ^2^) [77, 80]. Some readily-implementable methods for correcting for publication bias also exist, such as trim-and-fill methods [81, 82] or the use of the *p* curve [83]. The reader should be aware that these methods have shortcomings; for example, the trim-and-fill method can under- or overestimate an overall effect size, while the *p* curve probably only works when effect sizes come from tightly controlled experiments [83–86] (see Q9; note that ‘selection modeling’ is an alternative approach, but it is more technically difficult [79]). A less contentious topic in this area is the time-lag bias, where the magnitudes of an effect diminish over time [87–89]. This bias can be easily tested with a cumulative meta-analysis and visualized using a forest plot [90, 91] (Fig. 6d) or a bubble plot combined with meta-regression (Fig. 6e; note that journal impact factor can also be associated with the magnitudes of effect sizes [92], Fig. 6f).

Alarmingly, meta-reviews have found that only half of meta-analyses in ecology and evolution assessed publication bias [14, 15]. Disappointingly, there are no perfect solutions for detecting and correcting for publication bias, because we never really know with certainty what kinds of data are actually missing (although usually statistically non-significant and small effect sizes are underrepresented in the dataset; see also Q9). Regardless, the existing tools should still be used and the presentation of results from at least two different methods is recommended.

## Q9: Are results really robust and unbiased?

Although meta-analyses from the medical and social sciences are often accompanied by sensitivity analysis [69, 93], biological meta-analyses are often devoid of such tests. Sensitivity analyses include not only running meta-analysis and meta-regression without influential effect sizes or studies (for example, many effect sizes that come from one study or one clear outlier effect size; sometimes also termed ‘subset analysis’), but also, for example, comparing meta-analytic models with and without modeling non-independence (Q3–5), or other alternative analyses [44, 93]. Analyses related to publication bias could generally also be regarded as part of a sensitivity analysis (Q8). In addition, it is worthwhile checking if the authors discuss missing data [94, 95] (different from publication bias; Q8). Two major cases of missing data in meta-analysis are: 1) a lack of the information required to obtain sampling variance for a portion of the dataset (for example, missing standard deviations); and 2) missing information for moderators [96] (for example, most studies report the sex of animals used but a few studies do not). For the former, the authors should run models both with and without data with sampling variance information; note that without sampling variance (that is, unweighted meta-analysis) the analysis becomes a normal linear model [21]. For both cases 1 and 2, the authors could use data imputation techniques (as of yet, this is not standard practice). Although data imputation methods are rather technical, their implementation is becoming easier [96–98]. Furthermore, it may often be important to consider the sample size (the number and precision of constituent effect sizes) and statistical power of a meta-analysis. One of the main reasons to conduct meta-analysis is to increase statistical power. However, where an overall effect is expected to be small (as is often the case with biological phenomena) it is possible that a meta-analysis may be underpowered [99–101].

## Q10: Is the current state (and lack) of knowledge summarized?

In the discussion of a meta-analysis, it is reasonable to expect the authors to discuss what conventional wisdoms the meta-analysis has confirmed or refuted and what new insights the meta-analysis has revealed [8, 19, 71, 100]. New insights from meta-analyses are known as ‘review-generated evidence’ (as opposed to ‘study-generated evidence’) [18] because only aggregation of studies can generate such insights. This is analogous to comparative analyses bringing biologists novel understanding of a topic which would be impossible to obtain from studying a single species in isolation [14]. Because meta-analysis brings available (published) studies together in a systematic and/or comprehensive way (but see Q1), the authors can also summarize less quantitative themes along with the meta-analytic results. For example, the authors could point out what types of primary studies are lacking (that is, identify knowledge gaps). Also, the study should provide clear future directions for the topic under investigation [8, 19, 71, 100]; for example, what types of empirical work are required to push the topic forward. An obvious caveat is that the value of these new insights, knowledge gaps and future directions is contingent upon the answers to the previous nine questions (Q1–9).

## Post meta-evaluation: more to think about

Given that we are advocates of meta-analysis, we are certainly biased in saying ‘meta-analyses are enlightening’. A more nuanced interpretation of what we really mean is that meta-analyses are enlightening when they are done well. Mary Smith and Gene Glass published the first research synthesis carrying the label of ‘meta-analysis’ in 1977 [102]. At the time, their study and the general concept was ridiculed with the term ‘mega-silliness’ [103] (see also [16, 17]). Although the results of this first meta-analysis on the efficacy of psychotherapies still stand strong, it is possible that a meta-analysis contains many mistakes. In a similar vein, Robert Whittaker warned that the careless use of meta-analyses could lead to ‘mega-mistakes’, reinforcing his case by drawing upon examples from ecology [104, 105].

Even where a meta-analysis is conducted well, a future meta-analysis can sometimes yield a completely opposing conclusion from the original (see [106] for examples from medicine and the reasons why). Thus, medical and social scientists are aware that updating meta-analyses is extremely important, especially given that time-lag bias is a common phenomenon [87–89]. Although updating is still rare in biological meta-analyses [8], we believe this should become part of the research culture in the biological sciences. We appreciate the view of John Ioannidis who wrote, “Eventually, all research [both primary and meta-analytic] can be seen as a large, ongoing, cumulative meta-analysis” [106] (cf. effective thinking; Fig. 6d).

Finally, we have to note that we have just scratched the surface of the enormous subject of meta-analysis. For example, we did not cover other relevant topics such as multilevel (hierarchical) meta-analytic and meta-regression models [14, 45, 49], which allow more complex sources of non-independence to be modeled, as well as multivariate (multi-response) meta-analyses [107] and network meta-analyses [108]. Many of the ten appraisal questions above, however, are also relevant for these extended methods. More importantly, we believe that asking the ten questions above will readily equip biologists with the knowledge necessary to differentiate among mega-enlightenment, mega-mistakes, and something in-between.
